# The Resolution Approach to Cystic Fibrosis Inflammation

**DOI:** 10.3389/fphar.2020.01129

**Published:** 2020-07-29

**Authors:** Antonio Recchiuti, Sara Patruno, Roberto Plebani, Mario Romano

**Affiliations:** Laboratory of Molecular Medicine, Center on Advanced Studies and Technology (CAST), Department of Medical, Oral e Biotechnological Sciences, “G. d’Annunzio” University of Chieti-Pescara, Chieti, Italy

**Keywords:** inflammation resolution, specialized proresolving lipid mediators, ALX/FPR2 receptor, melanocortin system, melanocortin receptor (MCR), CFTR modulator therapy

## Abstract

Despite the high expectations associated with the recent introduction of CFTR modulators, airway inflammation still remains a relevant clinical issue in cystic fibrosis (CF). The classical anti-inflammatory drugs have shown very limited efficacy, when not being harmful, raising the question of whether alternative approaches should be undertaken. Thus, a better knowledge of the mechanisms underlying the aberrant inflammation observed in CF is pivotal to develop more efficacious pharmacology. In this respect, the observation that endogenous proresolving pathways are defective in CF and that proresolving mediators, physiologically generated during an acute inflammatory reaction, do not completely suppress inflammation, but promote resolution, tissue healing and microbial clearance, without compromising immune host defense mechanisms, opens interesting therapeutic scenarios for CF. In this mini-review, we present the current knowledge and perspectives of proresolving pharmacology in CF, focusing on the specialized proresolving lipid mediators and selected peptides.

## Introduction

Pulmonary inflammation and infection, leading to lung failure, represent the main cause of morbidity and mortality in individuals with cystic fibrosis (CF) (Cantin et al., 2015). Accumulating evidence indicates that loss-of-function of the Cystic Fibrosis Transmembrane Conductance Regulator (CFTR) is per se associated with a proinflammatory phenotype, even in the absence of infection (Khan et al., 1995). This is consistent with the observation that CFTR dysfunction primarily affects cells of the immune response including platelets, leukocytes and vascular endothelial cells (Painter et al., 2006; Mattoscio et al., 2010; Del Porto et al., 2011; Sorio et al., 2011; Plebani et al., 2017; Totani et al., 2017), making the pathogenesis of inflammation in CF quite complex. This may represent one of the reasons why the current anti-inflammatory pharmacology is of limited benefit to patients with CF.

With the advent of CFTR modulators, the CF therapeutic landscape has considerably changed. New highly effective modulator therapy combining one potentiator (ivacaftor) with two correctors (elexacaftor and tezacaftor) was recently tested in subjects carrying the F508del/F508del mutation, which causes the premature degradation of CFTR. Results from two randomized short-term trials are encouraging (Heijerman et al., 2019; Middleton et al., 2019), although the long-term impact of this treatment remains to be determined. Main question is whether modulators can significantly reduce the bacterial burden and ameliorate chronic inflammation. A partial answer to this question may be provided by a subsequent study of the GOAL trial, showing a downward trend in the relative abundance of *P. aeruginosa* and *S. aureus* in the airways of study participants treated with ivacaftor, but no changes in interleukin (IL)-6, -8, -1β, and free elastase in sputum (Rowe et al., 2014; Heltshe et al., 2015). Moreover, bacterial load in sputum of subjects treated with ivacaftor was reported to decline during the 1st year of treatment but started to increase afterwards (Hisert et al., 2017), suggesting that inflammation will eventually revamp as infection rebounds. Thus, it is still unclear whether CFTR modulators may downtone the inflammatory response sufficiently to prevent or slowdown the progression of lung deterioration. Consequently, therapies targeting inflammation continue to represent an important component of CF treatment.

Until recently, much of the research on CF airway phlogosis has been focused on the activation phase of the inflammatory response and has looked like a “boulevard of broken dreams” (Cantin et al., 2015). High dose ibuprofen is to date the only treatment of some efficacy to control CF inflammation and its short- and long-term beneficial effects have been confirmed by several studies (Konstan et al., 1995; Konstan et al., 2003; Konstan et al., 2018). However, ibuprofen can carry relevant side effects that limit its use for long periods in a large number of patients.

To avoid wrong assumptions and deleterious decisions, the quest for better drugs to combat CF inflammation should be grounded on an adequate knowledge of its pathogenetic mechanisms. In this respect, the experience with the leukotriene (LT)B_4_ receptor antagonist BIIL 284 gives a cautionary tale. Based on the strong evidence for the role of LTB_4_ in driving PMN lung infiltration and activation, a phase IIb/III clinical trial enrolling 600 adults and children with CF was conducted. Unfortunately, the trial had to be prematurely interrupted because of severe adverse effects in adults, including increase in pulmonary exacerbations (Konstan et al., 2014). A take-home message from this experience is that in developing anti-inflammatory agents for CF, we should keep in mind the characteristics of this disease, where an adequate anti-microbial immune response should be preserved. Therefore, an alternative, perhaps more rational, approach might be to enhance the body’s own mechanism to resolve inflammation.

## Specialized Proresolving Lipid Mediators

Pioneering work by Serhan and coworkers demonstrated that resolution of inflammation is an *active process* regulated by specific mediators, including a class of small lipid molecules termed specialized proresolving lipid mediators (SPM). SPM stop excessive PMN infiltration and activation, counter proinflammatory signals, enhance the active clearance of pathogens and dead cells by MΦ, are organ protective and stimulate tissue regeneration, thus accelerating the resolution of inflammation and restitutio ad integrum (reviewed by Serhan and Levy, 2018). SPM are biosynthesized from essential polyunsaturated fatty acids (PUFA) such as arachidonic acid (AA), eicosapentaenoic (EPA), docosapentaenoic (DPA) or docosahexaenoic acid (DHA). The SPM genus includes: lipoxins (LX) from AA, E-series resolvins (RvE) from EPA, D-series Rv, protectins (PD), and maresins (MaR) from DHA and their congenerous SPM from DPA (Recchiuti et al., 2019). Recently, SPM derived by the conjugation of epoxy-DHA to glutathione (GSH) have been uncovered and denominated “SPM conjugated in tissue regeneration” (Dalli et al., 2015).

The rationale for the use of SPM to control CF inflammation originates by studies showing that inflammation resolution is defective in CF, contributing to the development of lung disease. Karp et al. found reduced concentrations of LXA_4_ in BAL of CF children (Karp et al., 2004); our group demonstrated that CFTR loss-of-function dampens LX production during PLT:PMN interactions by a mechanism involving platelet 12-lipoxygenase (LO) dysfunction (Mattoscio et al., 2010). Defective LX-biosynthesis in CF was also recently reported (Ringholz et al., 2014). Along these lines, the RvD1:IL-8 ratio is diminished in sputum collected from individuals with CF compared to matched subjects without CF (Eickmeier et al., 2017; Isopi et al., 2020). In addition, we demonstrated that expression of ALX/FPR2, a receptor shared by LXA_4_ and RvD1, is significantly lower in F508del/F508del bronchial cells and CF MΦ (Pierdomenico et al., 2017). Remarkably, the reduced ALX/FPR2 expression blunts antimicrobial and proresolution responses of normal and CF cells to LXA_4_ and RvD1 (Pierdomenico et al., 2015; Pierdomenico et al., 2017).

Observation of defective SPM biosynthesis and downstream pathways in patients with CF provides the framework for innovative drugs that stimulate the generation of proresolving mediators in CF. Acebilustat (CTX-4430) is an oral inhibitor of LTA_4_ hydrolase that turns off LTB_4_ biosynthesis and increases LX formation. Results from phase I and II clinical trials with volunteers with mild to moderate CF, show that acebilustat significantly reduces sputum PMN number and neutrophil elastase levels in study participants (Elborn et al., 2017). A larger phase II trial has been conducted to identify the optimal patient population, dose, duration and endpoints for future acebilustat trials aimed at defining its efficacy in patients with CF (Elborn et al., 2018).

Lenabasum (JBT-101) is an oral agonist of leukocyte cannabinoid CB2 receptor that resolves experimental inflammation in mice by triggering LXA_4_ biosynthesis (Zurier et al., 2009). A phase IIa clinical trial of lenabasum has been recently completed in CF (Chmiel et al., 2017). Volunteers in the lenabasum arm had significant lower concentrations in sputum IL-8 and a downward trend in sputum neutrophil, elastase, and IgG, as well as in the risk of pulmonary exacerbation compared to volunteers in the no lenabasum arm. A multicenter phase IIb trial is underway (NCT03451045). In a recent study, lenabasum significantly reduced the number of PMN in exudate and level of proinflammatory prostanoids and increased the biosynthesis of RvD1 and LXA_4_ in human volunteers undergoing UV-killed *E. coli* skin injection (Motwani et al., 2018a).

SPM carry potent biological proresolving biactions. We reported that in wild type and *Cftr* knockout mice undergoing chronic *P. aeruginosa* infection, RvD1 reduces PMN influx shortening the time required to resolve inflammation, dampens bacterial load, and improves survival, weight recovery, and lung histopathology (Codagnone et al., 2018; Isopi et al., 2020). RvD1 also diminishes several cytokines and chemokines that are increased in CF airways including IL-8, IL-1β, and IL-17 and has additive effects when co-administered with ciprofloxacin at sub-optimal doses (Codagnone et al., 2018). Moreover, RvD1 enhances phagocytic clearance of *P. aeruginosa* in vivo and in vitro by human blood-derived and sputum MΦ and PMN from volunteers with CF (Isopi et al., 2020). In a mouse model of *P. aeruginosa* infection, Karp and coworkers also demonstrated that a LXA_4_ stable analog reduces PMN recruitment and bacterial titer (Karp et al., 2004), while other studies have shown that SPM reduces polymicrobial sepsis (Spite et al., 2009), peritonitis (Chiang et al., 2012), and pneumonia by viral and bacterial co-infection (Wang et al., 2017), indicating that counter-regulation of excessive inflammation and activation of host defense against pathogens are pivotal SPM bioactions.

Many of the actions exerted by SPM to limit inflammation and infection, such as the enhancement of bacterial phagocytosis by leukocytes (Chiang et al., 2012; Colas et al., 2014; Colas et al., 2016; Pierdomenico et al., 2017; Codagnone et al., 2018) and the ability to skew MΦ from a proinflammatory to a proresolutive phenotype (Dalli and Serhan, 2012; Recchiuti et al., 2014; Pistorius et al., 2018; Matte et al., 2019), were also recapitulated with isolated human cells. We recently demonstrated that RvD1 treatment of MΦ from volunteers with CF infected in vitro results in a broad modification of the transcriptomic fingerprint. In fact, RvD1 downregulated genes associated with inflammation, NF-κB activation, and leukocyte infiltration such as chemokines (CCL5, IL-8 and CXCL1), surface molecules (CD14, CD40, CD80, CCR5), PGE_2_ receptors (PTEGR) 2 and 4), and the 5-LO activating protein, which controls LTB_4_ synthesis and MΦ activation. On the contrary, RvD1 upregulated genes that enhance MΦ phagocytosis and reduce the inflammatory response, like CD93, IL10 receptor α (IL10RA), CD93, and the Wnt family member 1 and 7B (WNT1/7B) (Isopi et al., 2020).

SPM also act on airway epithelial cells regulating mucociliary clearance. LXA_4_ and RvD1 activate CFTR-independent Cl^-^ efflux and inhibit Na^+^ reabsorption, thus restoring the airway surface hydration (ASL) in CF bronchial epithelia (Verriere et al., 2012; Al-Alawi et al., 2014; Higgins et al., 2016; Ringholz et al., 2018). In airway epithelia exposed to bacterial infection in vitro, LXA_4_ and RvD1 also protect from cell injury, strengthen tight junctions and reduce IL-8 production (Grumbach et al., 2009; Higgins et al., 2016; Ringholz et al., 2018). In primary CF bronchial epithelial cells from F508del/F508del patients infected in vitro with *P. aeruginosa*, RvD1 upregulates the expression of genes that promote cell survival, such as tumor protein 63 (TP63), opioid receptor μ 1 (OPRM1), and aurora kinase B (AURKB), while it diminishes inflammatory genes, like CCL5 (Isopi et al., 2020).

SPM regulate inflammatory responses in the vasculature. LXA_4_ and B_4_ counter PMN chemotaxis triggered by LTB_4_ (Papayianni et al., 1996). RvD1 reduces PMN-EC interactions and transmigration (Sun et al., 2007; Norling et al., 2012) and diminishes vascular permeability induced by IL-1β and edema formation in vivo (Codagnone et al., 2018); LXA_4_ and RvD2 stimulate nitric oxide release that limits PMN adhesion to EC (Paul-Clark et al., 2004; Spite et al., 2009). Further, RvD4 modulates the formation of neutrophil extracellular traps that contribute to thrombosis and lung injury (Cherpokova et al., 2019), whereas RvE1 controls PLT/leukocyte interaction (Dona et al., 2008) and PLT aggregation (Fredman et al., 2010), which are dysregulated in people with CF and play significant pathogenetic roles in CF lung disease (Mattoscio et al., 2010; Ortiz-Muñoz et al., 2020).

Several clinical trials have demonstrated efficacy and safety of SPM in humans. In infants with eczema, a LXA_4_ stable analog was as potent as steroid treatment in reducing disease severity, eczema area and clinical scores (Wu et al., 2013). LXA_4_ proved superior efficacy to corticosteroids in improving lung function of asthmatic children (Kong et al., 2017). More recently, SPM stopped neutrophil infiltration in skin blisters raised in volunteers injected with UV-killed *E. coli* (Motwani et al., 2018b).

SPM act at multiple levels on cells and mechanisms involved in the pathophysiology of CF airway inflammation and activated resolution of inflammation and infection in preclinical and clinical studies, thus providing evidence for resolution pharmacology based on SPM in CF.

## Other Proresolving Agents Potentially Relevant for CF

### Annexin A1

Annexin A1 (ANXA1) is a calcium and phospholipid binding protein, induced by glucocorticoids that inhibits phospholipase A2 (Flower and Blackwell, 1979). ANXA1 is detectable in biological fluids and is widely expressed in both circulating (particularly PMN and monocytes) and resident (epithelial, endothelial and mesangial cells, fibroblasts and synoviocytes) cells from where it is released upon activation (reviewed by Sheikh and Solito, 2018). It promotes resolution by activating the ALX/FPR2 receptor (Perretti et al., 2002), shared with LXA_4_ and RvD1, placing this receptor at the crossroad of multiple proresolving pathways that can be altered in CF, where ALX/FPR2 expression is downregulated (Pierdomenico et al., 2017).

ANXA1 controls key proresolving mechanisms. It, in fact, limits PMN recruitment, while stimulating PMN apoptosis and clearance (reviewed by Sugimoto et al., 2016). It promotes M1 to M2 macrophage skewing (McArthur et al., 2020) and efferocytosis (Scannell et al., 2007). ANXA1 also downregulates proinflammatory cytokines and iNOS activity, while upregulating IL-10 expression (Ferlazzo et al., 2003). It stimulates tissue repair and reduces pulmonary fibrosis (Damazo et al., 2011). ANXA1 regulates platelet function in human and murine stroke, driving inflammation resolution (Senchenkova et al., 2019). This may be relevant in CF where platelet dysfunction drives lung hyperinflammation (Ortiz-Muñoz et al., 2020).

ANXA1 involvement in CF is documented by a number of reports. Downregulation of ANXA1 was observed in nasal epithelial cells from individuals with CF, as well as in lung and pancreas of *cftr ^-^/^-^* mice (Bensalem et al., 2005). Moreover, degradation of ANXA1 in bronchoalveolar lavage fluids from subjects with CF has been reported (Tsao et al., 1998). Consistent with these findings, administration of the selective CFTR inhibitor CFTRinh-172 to mice exacerbated zymosan-induced acute peritonitis, which was corrected by the administration of ANXA1 or its peptido mimetic (Dalli et al., 2010). More recently, the downregulation of ANXA1 was observed in injured tendon of F508del, thereby contributing to sustain inflammation (Liu et al., 2018). Therefore, targeting ANXA1 defects or supplying ANXA1 or its active peptide derivatives may be relevant to control CF inflammation.

### The Melanocortin System

The melanocortin system encompasses four peptide hormones, ACTH, α-MSH, β-MSH and γ-MSH, derived from post-translational processing of the precursor proopiomelanocortin (POMC), and two endogenous antagonists, agouti-related peptide (AgRP) and agouti signaling protein (ASIP) (Catania et al., 2004). ACTH is the best known melanocortin, because of its role in the hypothalamus-pituitary-adrenal axis and anti-inflammatory actions (Cone, 2006; Montero-Melendez, 2015).

Melanocortins activate five, high homologous, seven-transmembrane domains G protein-coupled receptors (MCR 1 to 5), some of which exert regulatory functions on the immune-inflammatory response (Patel et al., 2011). For instance, MCR1 is expressed by immune cells (monocytes, lymphocytes, neutrophils) (Brzoska et al., 2008) and carries anti-inflammatory and proresolution actions in ischemia-reperfusion (Leoni et al., 2008). MCR2 is activated only by ACTH and controls the synthesis of cortisol in the adrenal cortex (Xing et al., 2010), whereas MC3R has a relevant role in controlling lung inflammation (Getting et al., 2008) and ischemia-reperfusion (Leoni et al., 2008). MC5R is expressed also in immune cells and its activation is beneficial in immune disorders (Xu et al., 2020). Recently, we examined the proresolving signalling of MC1,3,4,5 receptors in human macrophages exposed to αMSH and some synthetic derivatives. ERK1/2 phosphorylation at any receptor was predominant to trigger efferocytosis and MC1R was the most relevant to downregulate cytokine release (Patruno et al., 2018).

The anti-inflammatory properties of the melanocortin system have been long known (reviewed by Wang et al., 2019). It is now recognized that ACTH exert proresolving actions, i.e. stimulation of efferocytosis, decrease in cytokine and chemokine accumulation, and increase in production of anti-inflammatory mediators, also independently by the hypothalamus-pituitary-adrenal circuit by targeting melanocortin receptors expressed by immune cells (Montero-Melendez, 2015). Moreover, similarly to SPM and ANXA1, melanocortins suppress the release of proinflammatory cytokines (Böhm et al., 1999; Patruno et al., 2018), inhibit PMN chemotaxis (Catania et al., 1996) and the NFkB pathway (Manna and Aggarwal, 1998). Melanocortins also inhibit the production of PGE_2_ (Nicolaou et al., 2004) and nitric oxide (Star et al., 1995), induce fibroblast senescence (Montero-Melendez et al., 2020) and reverse pulmonary fibrosis (Xu et al., 2011).

The melanocortin system exerts relevant protective action in the respiratory district (Moscowitz et al., 2019). α-MSH downregulates the MUC5AC-TNFα-NFkB pathway in nasal epithelial cells (Lee et al., 2011) and diminishes BAL infiltrate in allergic lung inflammation (Raap et al., 2003). Similar to SPM and ANXA1, it limits acute lung injury (Deng et al., 2004; Colombo et al., 2007).

Despite the promising outlook of the melanocortin system as endogenous machinery that, similarly to SPM, promotes inflammation resolution little is known regarding this system in CF. In a study of genome-wide association and linkage, Wright and co-workers reported that mutations in the MCR3 are associated with the severity CF lung disease (Wright et al., 2011), suggesting that MCR3 acts as a modifier gene in CF. A reasonable, yet to be tested, hypothesis could be that dysfunctions in the melanocortin system may contribute to sustain inflammation in CF and that pharmacological modulation of this system may downtone CF inflammation. Data from our laboratory seem to be in line with this hypothesis. We recently evaluated MCR expression and bioactions of α-MSH and a synthetic selective MC1R agonist, on macrophages and PMN from volunteers with CF. We consistently observed that these molecules exert anti-inflammatory (inhibition of cytokine release) and proresolving (stimulation of efferocytosis and PMN apoptosis) activities in addition to promoting microbial clearance (Patruno et al., 2019). Although preliminary, these results indicate that the melanocortin system may represent a promising field of investigation within the context of CF.

**Figure 1:**
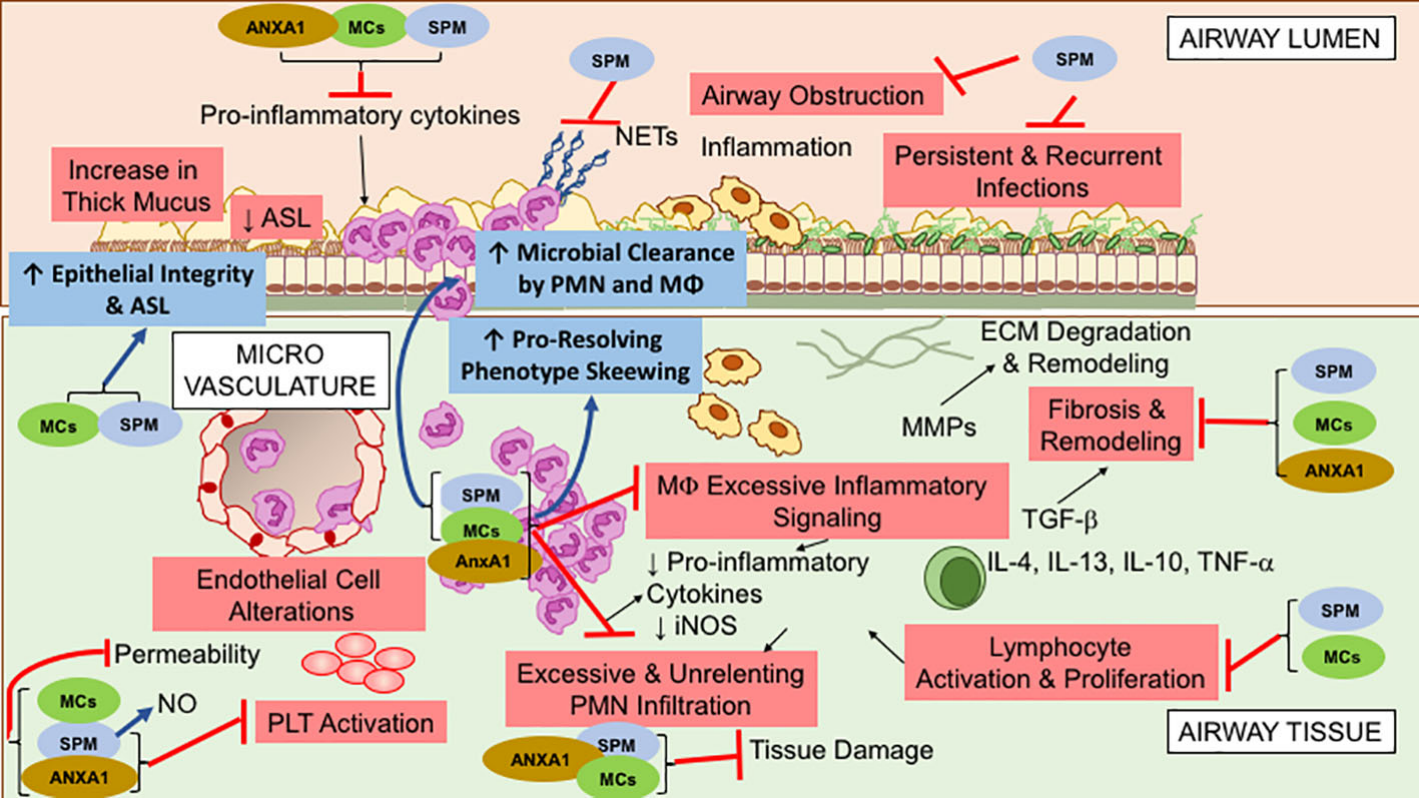
Overlapping specialized proresolving lipid mediators (SPM), Annexin A1(ANXA1), and Melanocortin (MCs) bioactions, relevant to control cystic fibrosis (CF) airway inflammation. These molecules exert multipronged functions, encompassing anti-inflammatory (limitation of further PMN infiltration, reduction in cytokine production, and decrease in lymphocyte, EC, and PLT activation) and proresolution (enhancement of MΦ phagocytosis and bacterial clearance, promotion of tissue repair, restoration of epithelial barrier integrity) activities. See within text and references for further details.

**Figure 1** shows the overlapping functions of SPM and proresolving peptides that are relevant in the pathogenesis of CF inflammation.

## Conclusion

The modest efficacy of the current anti-inflammatory pharmacology for CF lung disease reflects our incomplete knowledge of the mechanisms underlying the development of the aberrant inflammation that occurs in CF and its correlation with recurrent infection. Clinical and experimental evidence indicates that a complete suppression of the inflammatory response may be detrimental in CF, where instead reprogramming of the immune response to promote resolution appears to represent a more rational strategy. The discovery of endogenous proresolving pathways and the evidence that proresolving mediators promote resolution of inflammation and bacterial clearance in preclinical and in vitro models of CF opens new and promising perspectives for the development of innovate pharmacology for CF lung disease.

## Author Contributions

AR and MR conceived the manuscript. AR, SP, and RP wrote the manuscript. MR revised the manuscript. All authors contributed to the article and approved the submitted version.

## Funding

SP is supported by a fellowship from the American Cystic Fibrosis Foundation, Grant ROMANO19I0 to MR. AR is in part supported by grant L.548/93 from the Italian Ministry of Health, Regione Abruzzo, to MR.

## Conflict of Interest

The authors declare that the research was conducted in the absence of any commercial or financial relationships that could be construed as a potential conflict of interest.
